# AI-assisted versus point-based registration for digital planning in CBCT images with metallic artifacts

**DOI:** 10.1186/s12903-026-09990-0

**Published:** 2026-10-31

**Authors:** Nehal I. Shobair, Zainab Hafez Abdel Rahman, Mohamed Ahmed Mohamed Tolba, Yassin S. Alian

**Affiliations:** 1 Department of Oral and Maxillofacial Surgery, Faculty of Dentistry, Ain Shams University, Cairo, Egypt; 2 Department of Oral Medicine and Periodontology, Faculty of Dentistry, Misr International University, Cairo, Egypt; 3 Department of Histopathology, Faculty of Dentistry, Misr International University, Cairo, Egypt; 4 Department of Oral and Maxillofacial Prosthodontics, Faculty of Dentistry, Ain Shams University, Cairo, Egypt

**Keywords:** Artificial intelligence, Cone-beam computed tomography, Digital implant planning, Image registration, Guided implant surgery, Metallic artifacts

## Abstract

**Background:**

Accurate registration between cone-beam computed tomography (CBCT) images and intraoral scans is fundamental to digital implant planning because registration errors may propagate throughout the digital workflow and affect the accuracy of computer-guided implant surgery. Conventional point-based registration relies on manually selected anatomical landmarks and may be compromised by metallic artifacts that obscure dental structures. Artificial intelligence (AI)-assisted registration has recently emerged as a promising alternative; however, evidence comparing its performance with conventional point-based registration under clinically challenging conditions remains limited. This study aimed to compare the registration accuracy (primary outcome) and registration duration (secondary outcome) of AI-assisted and conventional point-based registration in patients presenting with metallic artifacts on CBCT images.

**Methods:**

This study included 45 eligible patients with corresponding full-arch CBCT datasets and intraoral scans. Registration accuracy was evaluated at the arch level by measuring the mean discrepancy at three predefined anatomical landmarks using Geomagic Control X software. Registration duration was recorded for each workflow. Arches were classified according to artifact burden into mild (> 0–33%), moderate (> 33–66%), and severe (> 66%) categories. Because the data were non-normally distributed, paired comparisons were performed using the Wilcoxon signed-rank test. Generalized estimating equations (GEE) were used as a sensitivity analysis to account for clustering of maxillary and mandibular arches within the same patient. Intra-observer reliability was assessed using the intraclass correlation coefficient (ICC).

**Results:**

A total of 78 paired arch observations were available for registration accuracy analysis. AI-assisted registration demonstrated significantly lower registration discrepancies than conventional point-based registration (median 0.190 mm [IQR 0.121–0.275] vs. 0.268 mm [IQR 0.200–0.418]; *p* < 0.001). Registration discrepancy increased with increasing artifact burden for both techniques, although AI-assisted registration consistently demonstrated lower discrepancies across all artifact severity groups. Registration duration was comparable between the two workflows. The GEE sensitivity analysis confirmed the primary findings after accounting for within-patient clustering. Intra-observer reliability was excellent (ICC = 0.985; 95% CI: 0.968–0.993).

**Conclusions:**

AI-assisted registration achieved significantly lower registration discrepancies than conventional point-based registration while maintaining comparable registration duration in CBCTs with metallic artifacts on CBCT images. These findings suggest that AI-assisted registration may improve the accuracy and consistency of multimodal image registration within the digital implant planning workflow, particularly in clinically challenging cases. Further prospective multicenter studies involving multiple implant-planning software platforms and broader clinical scenarios are required to confirm these findings.

## Introduction

Digital implant planning relies on the accurate integration of cone-beam computed tomography (CBCT) images with optical surface scans to create a virtual patient for computer-assisted implant surgery. Accurate multimodal image registration is fundamental because errors introduced during registration may propagate throughout the digital workflow and affect the accuracy of computer-guided implant surgery [1, 2].

Among the critical steps in this workflow are image segmentation and registration. Traditionally, segmentation has been performed manually, requiring considerable operator experience and substantial processing time [3]. To overcome these limitations, semi-automated segmentation techniques were introduced in which anatomical structures are identified using grayscale threshold values. Nevertheless, the quality of the segmented three-dimensional model remains highly dependent on CBCT image quality, directly influencing both segmentation and registration accuracy [1, 2].

Marker-free point-based registration (PBR) remains one of the most widely used techniques for aligning CBCT datasets with optical scans. This approach requires the manual identification of corresponding anatomical landmarks on both datasets to establish the initial alignment, after which surface-based registration is commonly performed to refine the superimposition [4–6]. Although this workflow has demonstrated acceptable accuracy, its performance depends on operator experience, the selection of reproducible anatomical landmarks, and the quality of the segmented CBCT model [7–9].

CBCT has become the imaging modality of choice for digital implant planning because of its ability to provide three-dimensional visualization of the maxillofacial skeleton. However, metallic restorations frequently generate beam-hardening and streak artifacts that obscure dental anatomy, distort the reconstructed surface, and reduce the availability of reliable anatomical landmarks required for image registration [4, 7]. Since conventional point-based registration and subsequent surface-based refinement depend on accurate segmentation and recognizable anatomical reference structures, metallic artifacts may adversely affect registration accuracy and ultimately influence the cumulative accuracy of guided implant surgery [6, 9].

Recent advances in artificial intelligence (AI), particularly deep learning, have enabled the development of automated image segmentation and registration algorithms. Unlike conventional workflows that rely primarily on manually selected landmarks, AI-assisted registration employs trained neural networks to automatically identify and align corresponding anatomical structures while reducing operator dependency [10, 11]. Similarly, deep-learning algorithms have substantially improved the accuracy and consistency of CBCT segmentation, providing more reliable three-dimensional models for digital workflows [12, 13]. In coDiagnostiX^®^, AI-assisted segmentation and image registration are performed through a cloud-based deep-learning service that automatically processes the uploaded CBCT data before registration, whereas the conventional workflow requires manual landmark selection followed by surface-based refinement [11].

Although previous studies have evaluated different CBCT–surface scan registration techniques [4–9, 14, 15], direct clinical comparisons between AI-assisted and conventional point-based registration in patients presenting with metallic artifacts remain limited. Therefore, the present retrospective clinical study aimed to compare the registration accuracy and registration duration of AI-assisted and conventional point-based registration in CBCT images with metallic artifacts. The null hypothesis was that no significant differences would exist between the two registration techniques regarding registration accuracy or registration duration.

## Materials and methods

### Study design

This study was conducted in accordance with the Strengthening Reporting of Observational Studies in Epidemiology (STROBE) guidelines. The study protocol was approved by the Research Ethics Committee of Misr International University (IRB No. MIU-IRB-2324-020) and prospectively registered at ClinicalTrials.gov (Identifier: NCT06273332, date of registration: 5 February 2024) before commencement of the data analysis. All participants had previously provided written informed consent permitting the use of their radiographic records for research purposes. The study was conducted in accordance with the ethical principles of the Declaration of Helsinki. All datasets were anonymized before analysis, and study-related information was securely stored on a password-protected computer accessible only to the principal investigator.

### Sample size calculation

An a priori sample size estimation was performed using G*Power software version 3.1.9.4 (Heinrich Heine University, Düsseldorf, Germany), based on the registration discrepancy values reported by Piao et al. [14]. Using the reported mean registration discrepancies of 0.29 ± 0.010 mm and 0.31 ± 0.009 mm, a two-sided significance level of 0.05 and statistical power of 95%, the initial calculation yielded a minimum total sample size of 16 observations. The reference values were used to estimate the magnitude of the anticipated between-method difference, while the two-sided hypothesis did not prespecify the direction of the effect. As this was a retrospective study, all cases meeting the predefined eligibility criteria during the specified study period were included, resulting in 45 patients contributing 78 paired arches with complete registration-accuracy measurements.

### Eligibility criteria

Previously acquired CBCT datasets and their corresponding intraoral scans were retrospectively retrieved from patients who fulfilled the predefined eligibility criteria.

Eligible cases included full-arch CBCT scans of the maxilla, mandible, or both presenting with one or more metallic restorations and a corresponding intraoral scan available in STL format. During CBCT acquisition, the maxillary and mandibular dentitions were separated to ensure clear visualization of the occlusal surfaces without overlapping or distortion.

Cases were excluded when CBCT images or intraoral scans were incomplete, required datasets were unavailable, image quality prevented reliable segmentation or registration. At the arch level, arches with fewer than five teeth available for application of the predefined five-landmark conventional point-based registration protocol were excluded.

### Study workflow

**Fig. 1:**
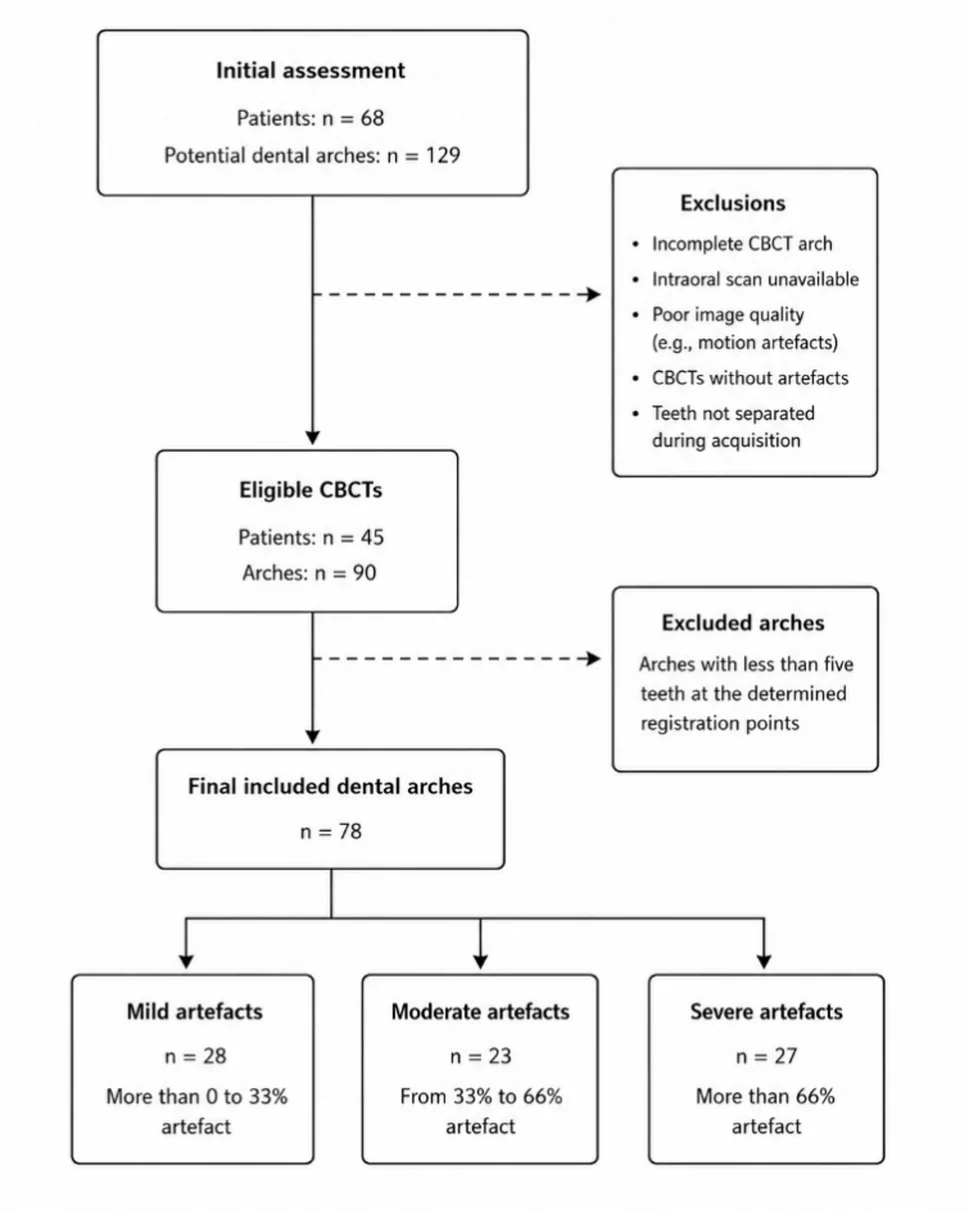
A Flowchart illustrating patient selection, application of the eligibility criteria, and allocation of the included dental arches according to metallic artifact severity

For each eligible patient, metallic restorations producing radiographic artifacts and the remaining natural teeth were counted separately for each arch. Artifact burden was calculated as the ratio of metallic artifacts to the remaining natural teeth for each arch. To facilitate clinically meaningful subgroup analyses, arches were classified according to artifact severity into three predefined categories: mild (> 0–33%), moderate (> 33–66%), and severe (> 66%). *From an initial cohort of 68 patients (129 potential dental arches)*, *45 patients (90 arches) met the eligibility criteria. After excluding arches with fewer than five teeth at the predefined registration landmarks*, *78 dental arches were included in the final analysis and categorized according to artifact burden into mild (> 0–33%;* *n = 28)*, *moderate (> 33–66%;* *n = 23)*, *and severe (> 66%;* *n = 27) artifact groups.*( Fig. 1).

**Fig. 2:**
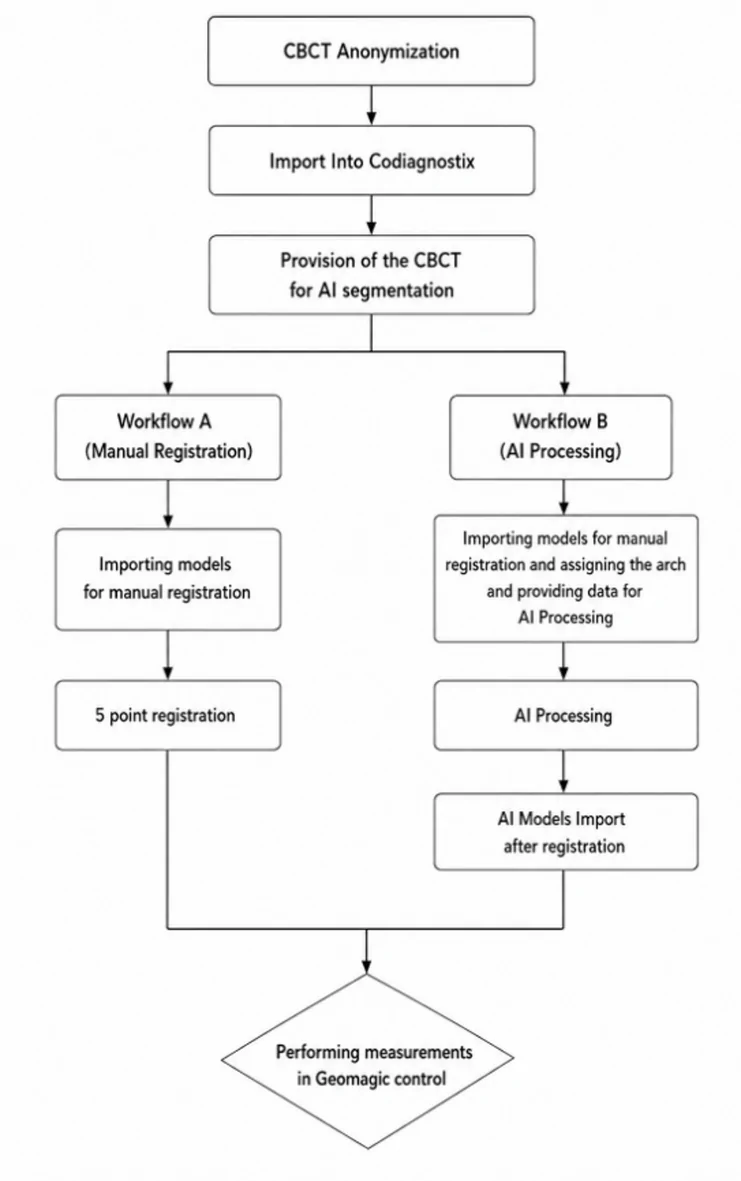
Study workflow illustrates the registration procedures evaluated in the present study

Figure 2 demonstrated the digital workflow of the study. All CBCT datasets were segmented using the artificial intelligence-assisted segmentation module incorporated within coDiagnostiX^®^ software (Version 10.7; Dental Wings Inc., Montreal, Canada). Segmentation was performed by an independent investigator using a standardized protocol for all datasets. The same segmented CBCT model was subsequently used for both registration methods to ensure that any observed differences reflected the registration procedure rather than the segmentation process itself. Following segmentation, each dataset underwent both conventional point-based registration and AI-assisted registration.

**Fig. 3:**
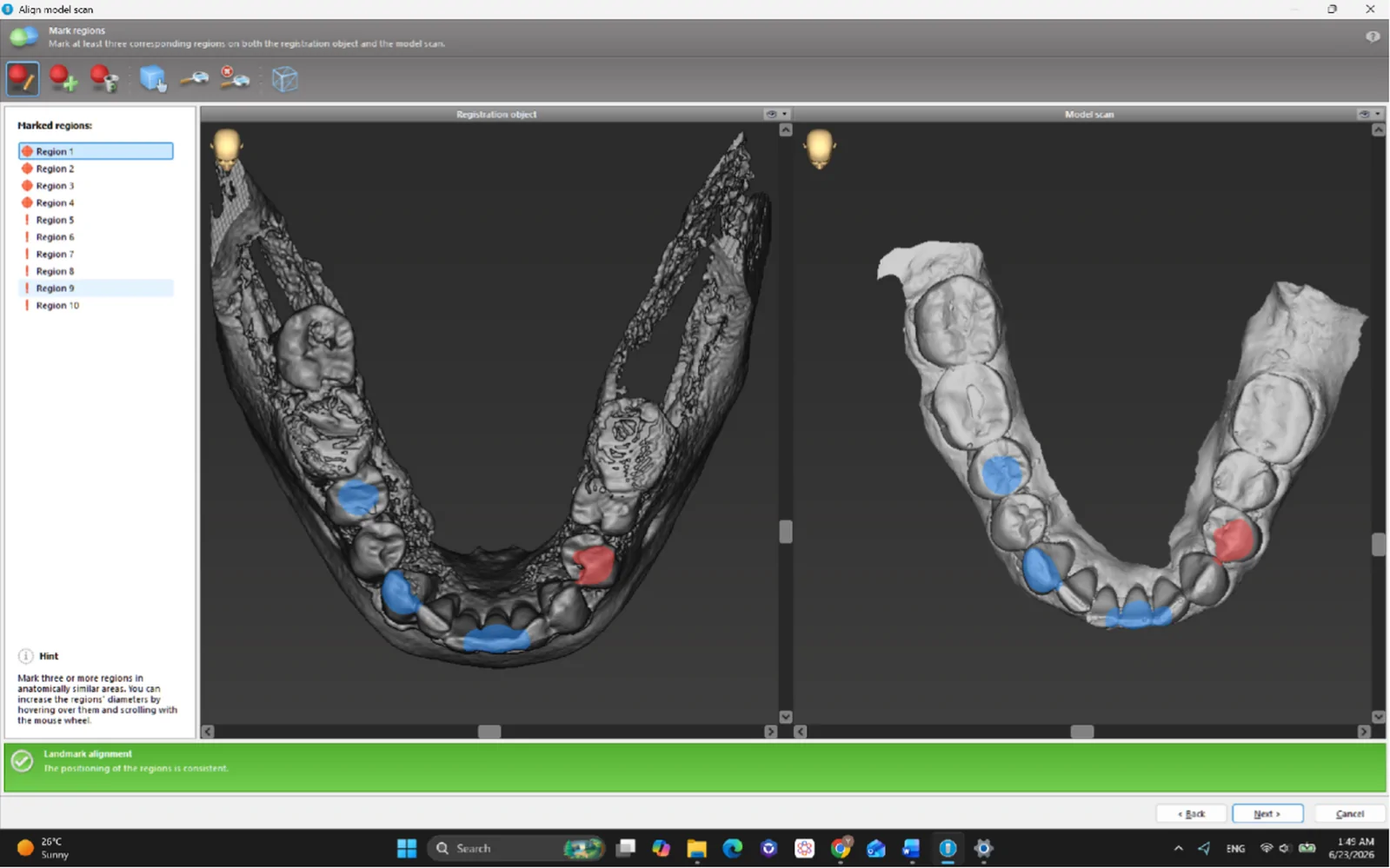
Conventional point-based registration workflow in coDiagnostiX^®^ software

**Fig. 4:**
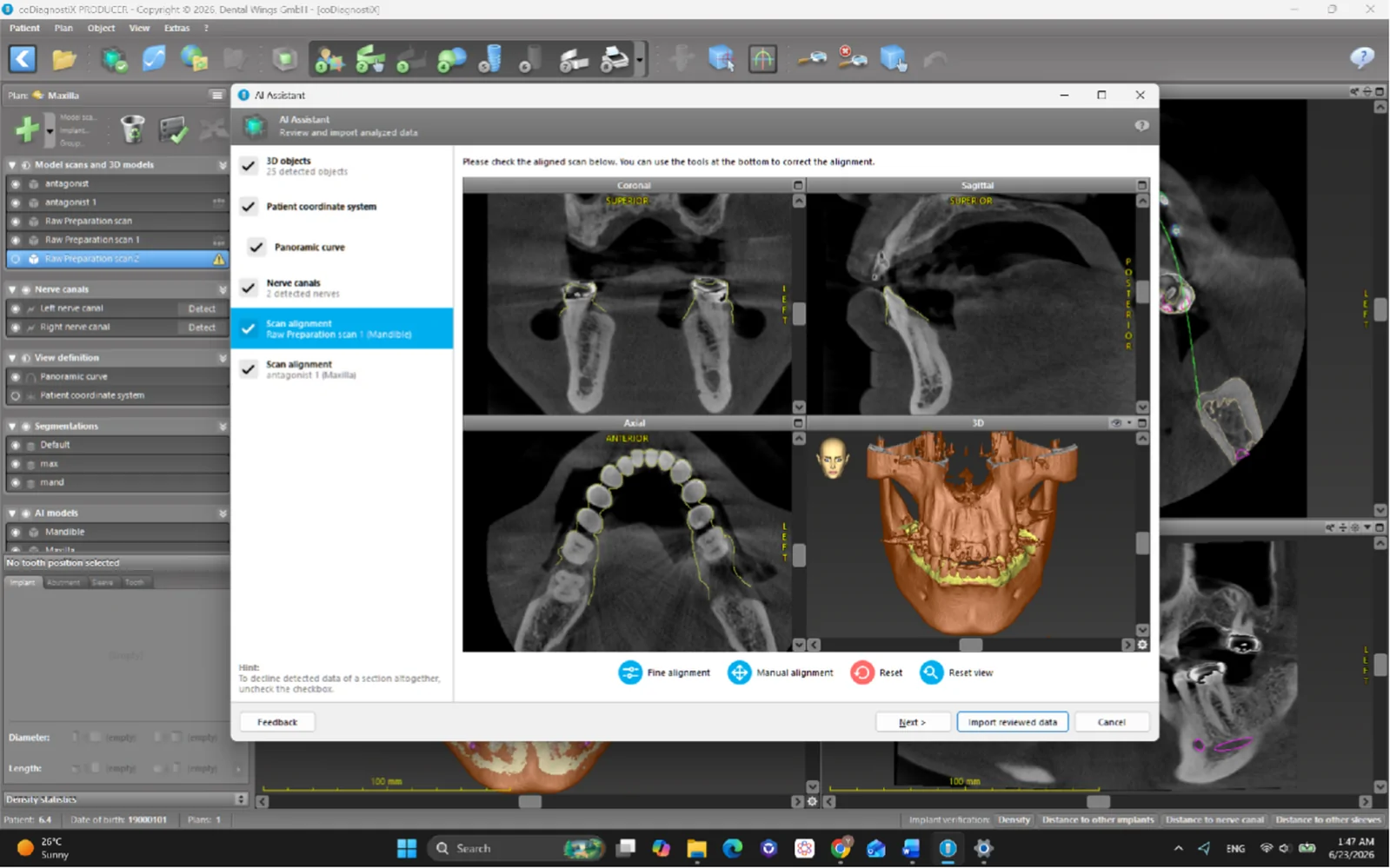
AI-assisted registration workflow in coDiagnostiX^®^ software

Conventional point-based registration was performed according to the protocol described by Park et al. [8]. Five corresponding anatomical landmarks were manually identified on both the CBCT-derived model and the intraoral scan: the midpoint of the incisal edges of the central incisors, the bilateral cusp tips of the permanent canines, and the bilateral mesiobuccal cusp tips of the first permanent molars. Following initial landmark alignment, automated surface-based refinement was performed by the software to optimize the final registration. AI-assisted registration was subsequently performed using the automated registration workflow integrated within coDiagnostiX^®^. (Fig. 3) The AI-assisted workflow automatically prepared the datasets and identified anatomical features to facilitate multimodal image registration while reducing manual operator interaction. (Fig. 4) Following visual verification and acceptance of the final alignment, all registered datasets were exported in the coDiagnostiX^®^ patient coordinate system and securely stored for subsequent analysis.

### Registration accuracy assessment

**Fig. 5:**
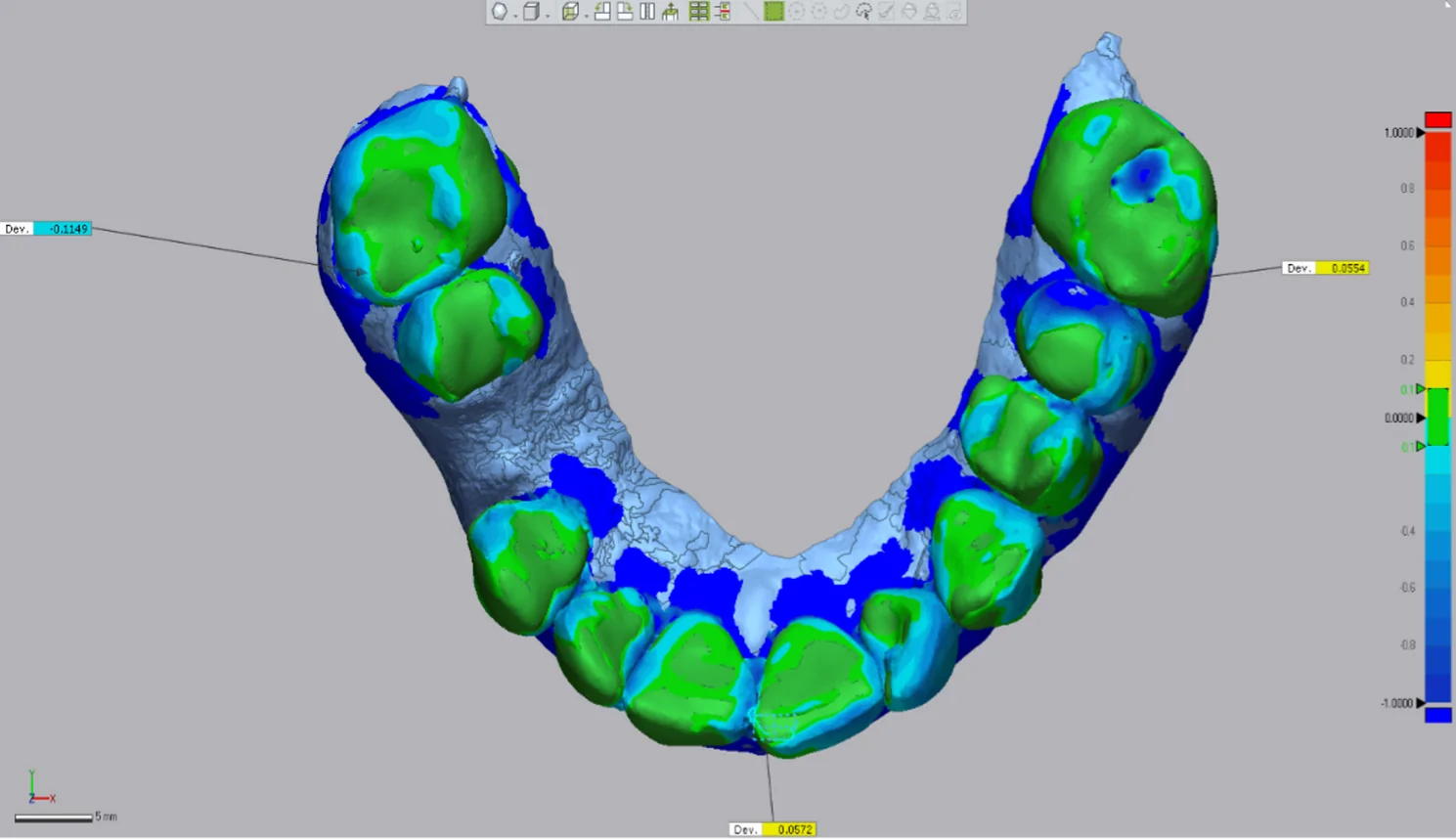
Representative assessment of registration discrepancy in Geomagic Control X software. The color-coded deviation map demonstrates the alignment between the registered CBCT-derived dental model and the corresponding intraoral scan

Registration accuracy was evaluated at the arch level. For each registered arch, three predefined dental landmarks were selected to represent the anterior and bilateral posterior regions of the dental arch. These landmarks consisted of the mesial point angle of the central incisor and the cusp tips of the most posterior standing teeth on the right and left sides. Absolute linear discrepancy (gap distance) between the registered intraoral scan and the corresponding CBCT-derived model was measured at each landmark using Geomagic Control X 2020 software (3D Systems, North Carolina, USA). (Fig. 5) Measurements were recorded in millimeters. The mean of the three landmark measurements represented the registration discrepancy for each individual arch.

### Registration duration assessment

Registration duration was measured using a digital stopwatch throughout the registration workflow. Timing included model import & registration. For the AI-assisted workflow, file upload and download intervals required for cloud-based processing were recorded separately and excluded from the reported registration duration because these intervals depended primarily on internet connection speed, network latency, file size, and server communication rather than the registration algorithm itself.

### Statistical analysis

Statistical analyses were performed using IBM SPSS Statistics for Windows, Version 24.0 (IBM Corp., Armonk, NY, USA). Continuous variables were assessed for normality using the Shapiro–Wilk and Kolmogorov–Smirnov tests. As the data were not normally distributed, continuous variables are presented as median and interquartile range (IQR) as the primary descriptive statistics, with mean ± standard deviation (SD) reported for descriptive purposes. Registration accuracy was analyzed at the arch level, with each maxillary and mandibular arch considered an individual observational unit. Registration discrepancy was calculated as the mean of three predefined landmark measurements for each arch. The primary outcome, registration accuracy, was compared between AI-assisted registration and conventional point-based registration using the Wilcoxon signed-rank test. Registration duration was analyzed using the same test. Effect sizes were calculated for all paired comparisons which were interpreted as small ($\:r\ge\:0.10$), moderate ($\:r\ge\:0.30$), and large ($\:r\ge\:0.50$).

To investigate the influence of metallic artifact burden on registration accuracy, arches were categorized into three predefined groups according to the artifact-to-natural tooth ratio: mild (0–33%), moderate (> 33–66%), and severe (> 66%). Registration discrepancies within these subgroups were summarized descriptively. The association between artifact burden and registration discrepancy was evaluated using Spearman’s rank correlation coefficient (ρ). Correlation analyses involving maxillary and mandibular arches were considered exploratory and were interpreted cautiously because multiple comparisons were performed.

Because maxillary and mandibular arches originated from the same patient and therefore were not statistically independent, a generalized estimating equations (GEE) analysis with robust standard errors was performed as a sensitivity analysis to account for within-patient clustering and to confirm the robustness of the primary findings.

Intra-observer reliability was assessed by repeating registration accuracy measurements for 30 randomly selected arches after a two-week interval. Reliability was evaluated using the Intraclass Correlation Coefficient (ICC) based on a two-way mixed-effects model with absolute agreement for single measurements. The 95% confidence interval (CI), mean difference between repeated measurements, standard deviation of the differences, and root mean square error (RMSE) were calculated to quantify measurement reproducibility and systematic error. All statistical tests were two-sided, and statistical significance was established at *p* < 0.05.

### Study population

A total of 45 eligible patients were included in this study. Registration accuracy was evaluated at the arch level, resulting in 78 paired arch observations with complete registration accuracy measurements for both registration techniques.

### Intra-observer reliability

Intra-observer reliability analysis demonstrated excellent agreement between the initial and repeated measurements. The intraclass correlation coefficient was 0.985 (95% CI: 0.968–0.993), indicating excellent reproducibility of the registration accuracy measurements. The mean difference between repeated measurements was 0.0097 mm, with a standard deviation of 0.0313 mm and a root mean square error (RMSE) of 0.0323 mm, demonstrating minimal systematic measurement error.

### Registration accuracy

**Fig. 6:**
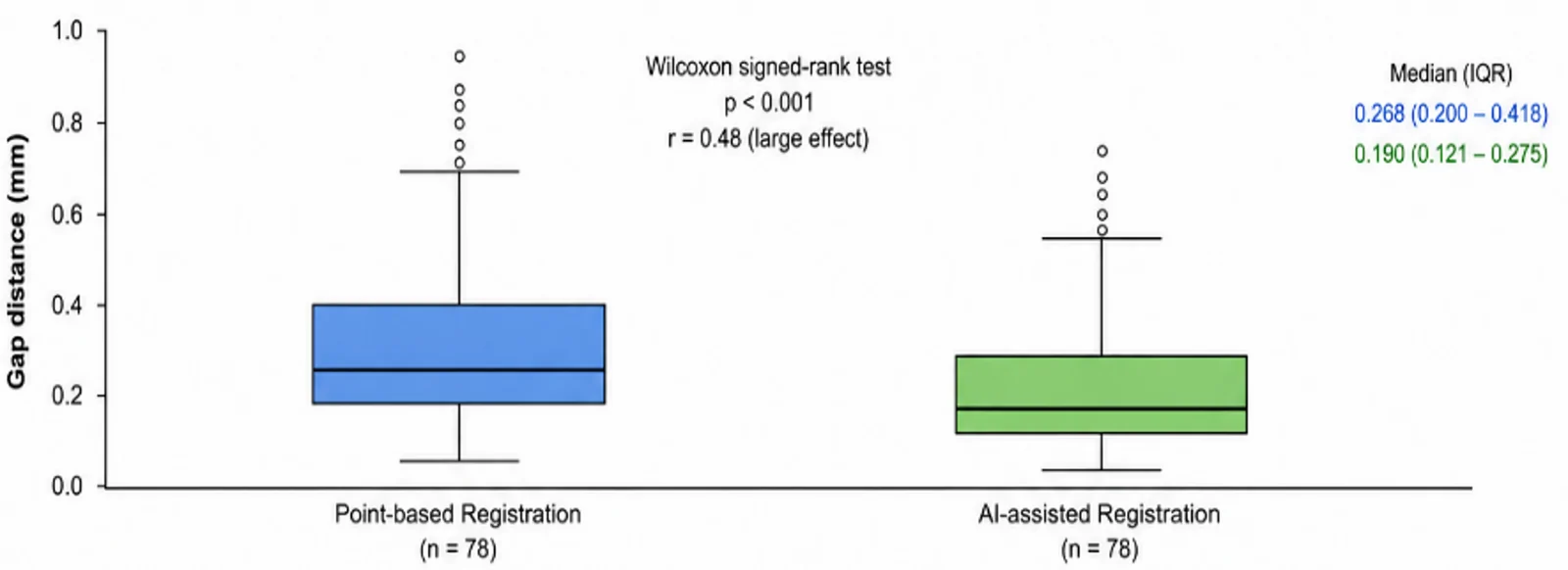
Boxplots comparing registration discrepancy between conventional point-based and AI-assisted registration. Registration discrepancy was quantified as the gap distance (mm) between the registered CBCT-derived three-dimensional model and the corresponding intraoral scan

AI-assisted registration demonstrated significantly lower registration discrepancies than conventional point-based registration. The median registration discrepancy was 0.190 mm (IQR: 0.121–0.275 mm) for the AI-assisted registration group compared with 0.268 mm (IQR: 0.200–0.418 mm) for the point-based registration group. Mean registration discrepancies were 0.245 ± 0.199 mm and 0.392 ± 0.393 mm, respectively (Table 1) (Fig. 6). The paired comparison demonstrated a statistically significant reduction in registration discrepancy using AI-assisted registration compared with conventional point-based registration (Wilcoxon signed-rank test, *p* < 0.001), indicating improved registration accuracy with the AI-assisted workflow.

**Table 1:** Registration accuracy and registration duration

Outcome	Point-based Registration	AI-assisted Registration	Wilcoxon W	p- value	effect size r
Registration accuracy (mm)					
Number of arches, n	78	78			
Median (IQR)	0.268 (0.200–0.418)	0.190 (0.121–0.275)	686	< 0.001	0.482
Mean ± SD	0.392 ± 0.393	0.245 ± 0.199			
Minimum–Maximum	0.056–2.399	0.036–1.204			
Registration duration (seconds)					
Number of arches, n	78	78			
Median (IQR)	131.0 (121.0–154.5)	148.1 (122.6–162.4)	1562	0.519	0.071
Mean ± SD	143.9 ± 39.0	148.9 ± 57.7			
Minimum–Maximum	100–380	46.5–453.4			

### Registration duration

**Fig. 7:**
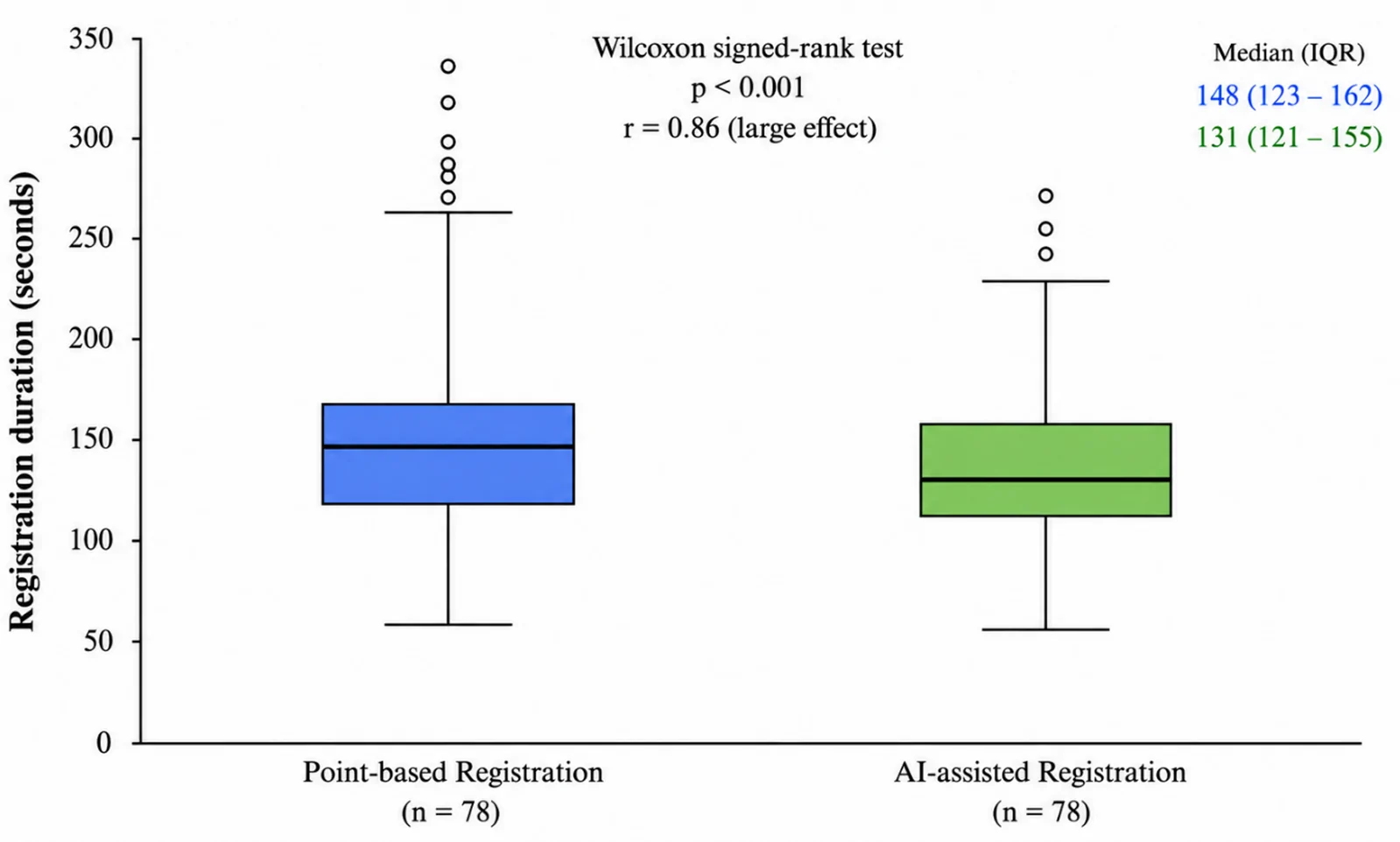
Boxplots comparing registration duration between conventional point-based and AI-assisted registration. Registration duration (seconds) was recorded for each registration workflow

Registration duration was comparable between the two registration techniques. The median registration duration was 131.0 s (IQR: 121.0–154.5 s) for conventional point-based registration and 148.1 s (IQR: 122.6–162.4 s) for AI-assisted registration. Mean registration durations were 143.9 ± 39.0 s and 148.9 ± 57.7 s, respectively (Table 1) (Fig. 7). Although AI-assisted registration required a slightly longer median active registration time, the difference between the two techniques was not statistically significant. The maximum AI-assisted registration duration of 453.4 s occurred in a full-skull CBCT dataset that required repeated import attempts before successful completion of the registration workflow. Review of the timing record confirmed that this value did not include network-dependent upload or download time.

Registration accuracy according to artifact severity. For the AI-assisted workflow, the time required for cloud-dependent file transfer was recorded separately and excluded from the active registration duration. The network-dependent file-transfer interval was analyzed separately from the active registration duration. The mean combined upload and download time for the CBCT and model files was 119.73 ± 26.07 s under an internet connection with measured download and upload speeds of approximately 426.0 Mbps and 38.7 Mbps, respectively.

**Fig. 8:**
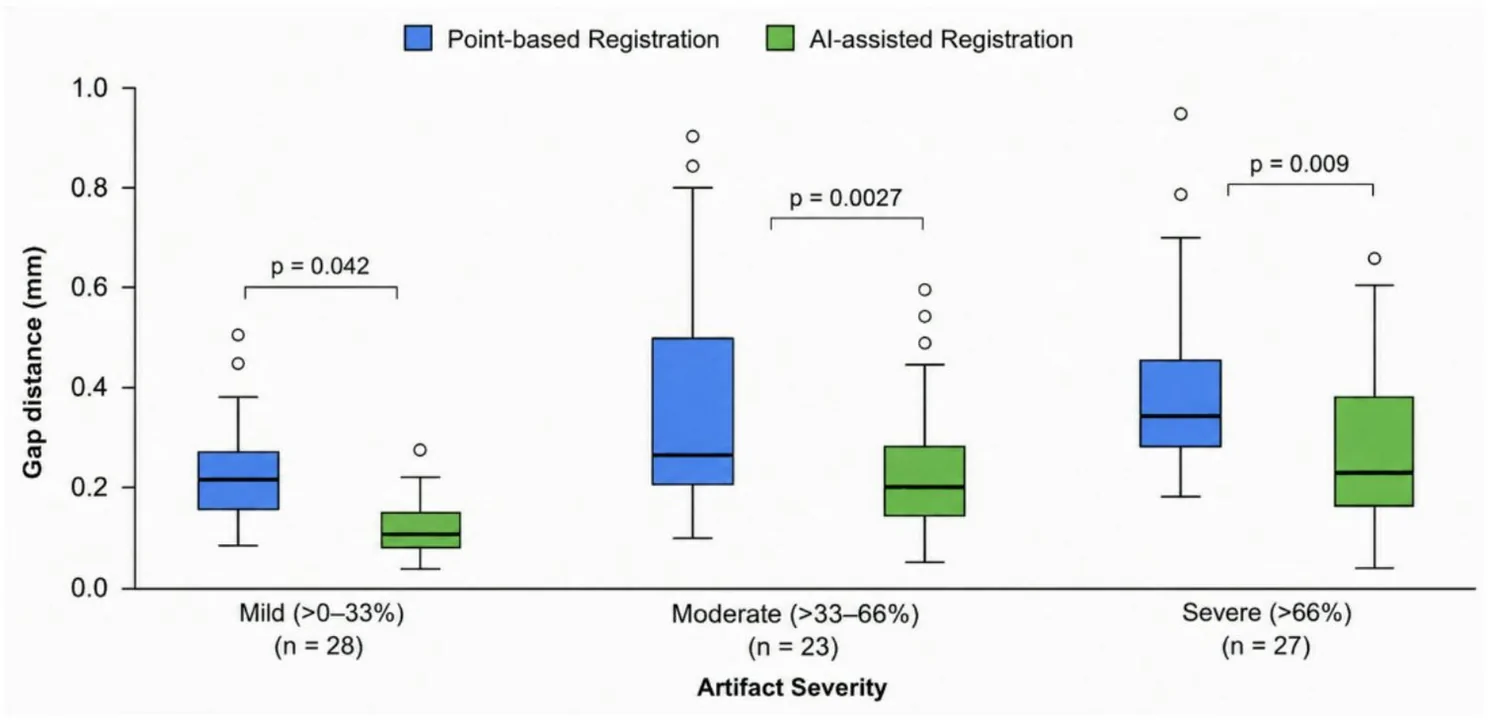
Boxplots comparing registration discrepancy according to metallic artifact severity for conventional point-based and AI-assisted registration. Registration discrepancy (gap distance, mm) is presented for the mild (> 0–33%), moderate (> 33–66%), and severe (> 66%) metallic artifact groups. Each box represents the interquartile range (IQR), the horizontal line indicates the median, whiskers represent 1.5 × IQR, and circles denote outliers. Comparisons between registration techniques within each artifact severity group were performed using the Wilcoxon signed-rank test

Registration discrepancies increased progressively with increasing artifact burden for both registration techniques (Table 2) (Fig. 8). Across all artifact severity categories, AI-assisted registration demonstrated lower median registration discrepancies than conventional point-based registration. For arches with mild artifact burden (> 0–33%), median registration discrepancies were 0.210 mm for point-based registration and 0.082 mm for AI-assisted registration. In the moderate artifact group (> 33–66%), median discrepancies were 0.249 mm and 0.190 mm, respectively. In arches with severe artifact burden (> 66%), median registration discrepancies increased to 0.337 mm for point-based registration and 0.220 mm for AI-assisted registration.

**Table 2:** Registration accuracy according to artifact severity

Artifact severity	*n*	Point-based Registration Median (IQR), mm	AI-assisted Registration Median (IQR), mm
Mild (> 0–33%)	28	0.210 (0.110–0.264)	0.082 (0.043–0.166)
Moderate (> 33–66%)	23	0.249 (0.191–0.530)	0.190 (0.130–0.270)
Severe (> 66%)	27	0.337 (0.277–0.446)	0.220 (0.157–0.384)

Artifact severity was classified according to the artifact-to-natural tooth ratio as mild (> 0–33%), moderate (> 33–66%), and severe (> 66%). IQR, interquartile range

### Correlation between artifact burden and registration discrepancy

**Fig. 9:**
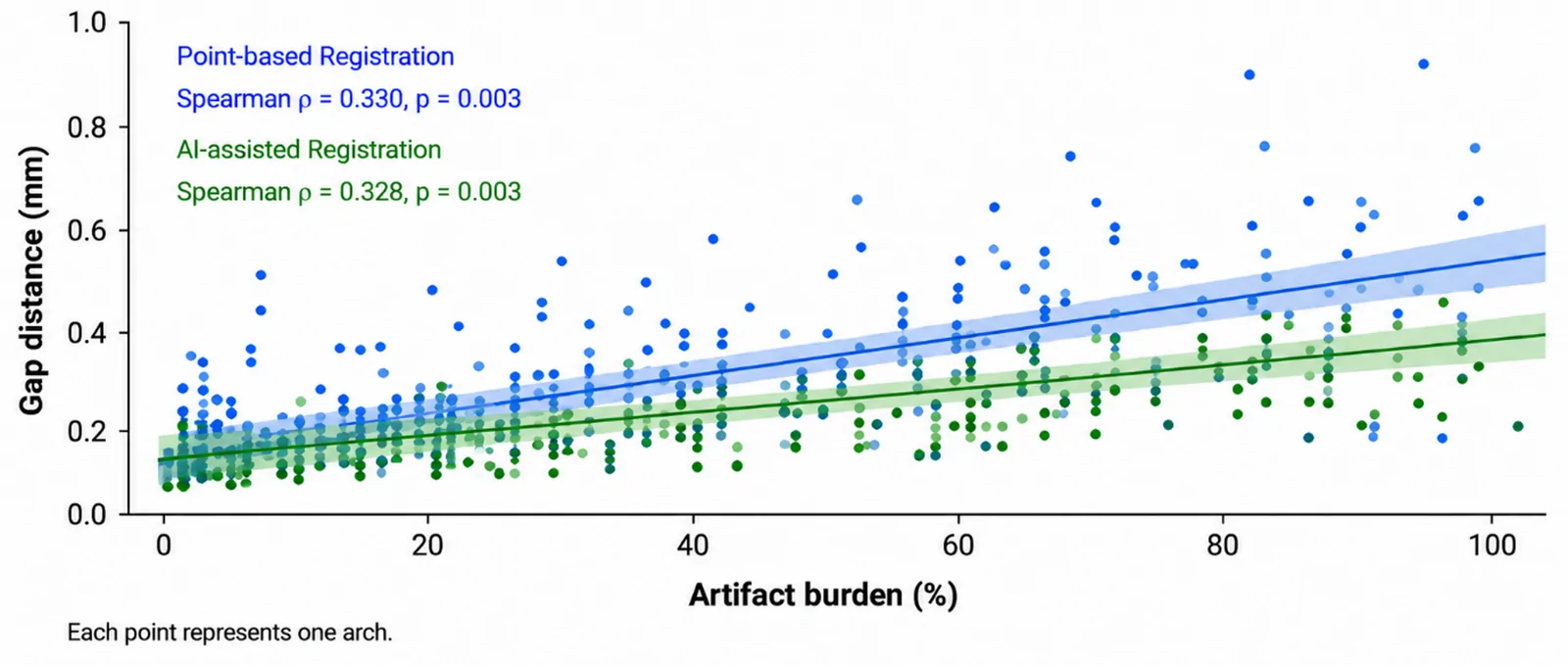
Relationship between metallic artifact burden and registration discrepancy for conventional point-based and AI-assisted registration. Scatter plots with fitted linear regression lines illustrate the association between artifact burden (%) and registration discrepancy (gap distance, mm) for both registration techniques. The shaded areas represent the 95% confidence intervals of the fitted regression lines. Correlations were assessed using Spearman’s rank correlation coefficient (ρ), with each point representing one dental arch

A weak but statistically significant positive correlation was observed between artifact burden and registration discrepancy for both registration techniques (Table 3) (Fig. 9). For conventional point-based registration, Spearman’s correlation coefficient was ρ = 0.330 (*p* = 0.003), while AI-assisted registration demonstrated a similar correlation (ρ = 0.328, *p* = 0.003). These findings indicate that registration discrepancy increased with increasing artifact burden regardless of the registration technique.

**Table 3:** Correlation between artifact burden and registration discrepancy

Registration technique	Spearman’s correlation coefficient (ρ)	*p*-value	Interpretation
Point-based registration	0.33	0.003	Weak positive correlation
AI-assisted registration	0.328	0.003	Weak positive correlation

Spearman’s rank correlation coefficient was used to assess the association between artifact burden (artifact-to-natural tooth ratio) and registration discrepancy. Positive correlation coefficients indicate increased registration discrepancy with increasing artifact burden.

### Sensitivity analysis

A generalized estimating equations (GEE) analysis was performed to account for clustering of maxillary and mandibular arches within individual patients. The GEE model confirmed the primary paired analysis, demonstrating that point-based registration was associated with significantly greater registration discrepancy than AI-assisted registration (β = 0.147, 95% CI: 0.066–0.229, *p* < 0.001) (Table 4). These findings support the robustness of the primary results after accounting for within-patient dependence.

**Table 4:** Generalized estimating equations (GEE) sensitivity analysis of registration discrepancy

Variable	β coefficient	Standard error	95% Confidence Interval	*p*-value
Intercept (AI-assisted registration)	0.245	0.024	0.198–0.292	< 0.001
Point-based registration (vs. AI-assisted)	0.147	0.042	0.066–0.229	< 0.001

## Discussion

The present retrospective observational study compared the registration accuracy and registration duration of AI-assisted and conventional point-based registration in CBCT images with metallic artifacts. Registration accuracy was evaluated at the arch level, reflecting the clinical workflow in which each arch undergoes an independent registration procedure. The primary finding of the present study was that AI-assisted registration achieved significantly lower registration discrepancies than conventional point-based registration while maintaining comparable registration duration. In addition, registration discrepancy increased with increasing metallic artifact burden regardless of the registration technique, although AI-assisted registration consistently demonstrated lower discrepancies across all artifact severity categories. These findings remained consistent after accounting for clustering of maxillary and mandibular arches using generalized estimating equations, supporting the robustness of the primary analysis.

Accurate registration between CBCT datasets and intraoral scans is a fundamental prerequisite for digital implant planning because it establishes the spatial relationship between anatomical structures and the prosthetic surface model used throughout virtual treatment planning. Registration errors introduced at this stage contribute to cumulative inaccuracies that may occur during subsequent steps of the digital workflow, including virtual treatment planning, surgical guide fabrication, and implant placement [1, 2, 4]. Consequently, improving registration accuracy should be viewed as optimizing one critical component of the digital workflow rather than as an isolated procedural outcome. This concept becomes particularly relevant in patients presenting with metallic restorations, where image degradation increases the complexity of multimodal image registration [7, 9].

The present study demonstrated a statistically significant reduction in registration discrepancy with AI-assisted registration compared with conventional point-based registration. Although the observed differences were within the submillimeter range, image registration represents only one component of the cumulative digital workflow. Therefore, improvements in registration accuracy should be interpreted as reducing one source of cumulative workflow error rather than directly improving implant placement accuracy. Systematic reviews evaluating the accuracy of static computer-guided implant surgery have consistently demonstrated that the final positional deviation results from the accumulation of inaccuracies introduced during CBCT acquisition, intraoral scanning, image registration, virtual treatment planning, surgical guide manufacturing, and surgical execution rather than from a single procedural step [16, 17]. Accordingly, although the absolute reduction in registration discrepancy observed in the present study was modest, AI-assisted registration may improve the reliability and predictability of digital implant planning, particularly in clinically challenging situations characterized by extensive metallic restorations.

The present findings are consistent with previous investigations demonstrating the importance of accurate multimodal image registration for digital implant planning. Piao et al. (2022) [14] reported that both registration technique and the presence of dental restorations influence the accuracy and efficiency of CBCT–intraoral scan registration, emphasizing the importance of selecting an appropriate registration strategy in clinically challenging situations. More recently, Elgarba et al. (2024) [11] demonstrated that AI-assisted CBCT–intraoral scan registration achieved accurate and reproducible multimodal alignment while substantially reducing operator interaction, supporting the clinical feasibility of automated registration workflows. Similarly, Chung et al. (2020) [10] showed that deep learning-based registration algorithms can accurately align volumetric CBCT images and optical surface scans by learning complex anatomical relationships between the two imaging modalities. Unlike these previous investigations, the present study specifically evaluated AI-assisted and conventional point-based registration in patients presenting with metallic artifacts, a clinical scenario in which image degradation may substantially compromise registration accuracy. Therefore, the present findings extend the current evidence by demonstrating the performance of AI-assisted registration under artifact-compromised clinical conditions.

One of the most clinically relevant findings of the present study was the influence of metallic artifact burden on registration performance. Metallic restorations produce beam-hardening and scatter artifacts that obscure anatomical landmarks, distort tooth morphology, and reduce CBCT image quality, thereby increasing the complexity of multimodal image registration [4, 7]. Consistent with this phenomenon, registration discrepancy demonstrated a weak but statistically significant positive correlation with increasing artifact burden for both registration techniques. Nevertheless, AI-assisted registration consistently demonstrated lower registration discrepancies across all artifact severity groups, suggesting greater robustness under challenging imaging conditions. One possible explanation is that the advantage arises from the automated registration algorithm, which identifies and aligns complex anatomical patterns after segmentation rather than relying solely on manually selected anatomical landmarks, potentially making the registration process less susceptible to localized image degradation caused by metallic artifacts [10, 11]. However, because the subgroup analyses were exploratory and the study was not specifically powered for subgroup comparisons, these findings should be interpreted cautiously and require confirmation in larger prospective studies.

Registration duration constituted the secondary outcome of the present study. Despite the additional automated processing incorporated into the AI-assisted workflow, registration duration remained comparable between AI-assisted and conventional point-based registration. To ensure that this comparison reflected the intrinsic efficiency of the registration methods rather than external technical factors, network-dependent upload and download intervals were intentionally excluded from the AI-assisted workflow. In the present study, conventional point-based registration was performed locally within the coDiagnostiX^®^ software environment, whereas AI-assisted segmentation and registration required communication with a remote cloud-based AI service [18]. The mean combined upload and download time was 119.73 ± 26.07 s under relatively high-speed network conditions (approximately 426.0 Mbps download and 38.7 Mbps upload). Because these transfer intervals are primarily influenced by infrastructure-related factors, including internet bandwidth, server communication, and file transfer performance, rather than by the registration algorithm itself, they were excluded from the active registration-duration comparison to minimize variability unrelated to the registration process [11]. Nevertheless, network-dependent transfer time remains part of the total elapsed clinical workflow and may have a greater impact on the practical efficiency of cloud-based AI-assisted registration in settings with lower bandwidth, higher latency, or less stable internet connectivity.

An additional distinction between the two workflows was the nature of operator involvement. Conventional point-based registration consisted almost entirely of active operator-dependent time, requiring continuous user interaction, anatomical landmark identification, and operator expertise throughout the registration procedure. In contrast, the AI-assisted workflow required only brief operator input to initiate processing, after which segmentation and registration proceeded automatically without further user intervention [11, 14]. During this passive processing phase—including data upload, cloud-based computation, and retrieval of the processed results—the operator was free to perform other clinical or laboratory tasks, making these intervals fundamentally different from the active working time required for conventional point-based registration. Accordingly, AI-assisted registration may improve workflow efficiency in clinical practice by reducing active operator involvement without increasing overall registration time [2]. Nevertheless, because upload and download intervals remain part of the real-world clinical workflow, they should be considered when interpreting the practical efficiency of cloud-based AI registration systems.

Several methodological considerations strengthen the interpretation of the present findings. First, registration accuracy was evaluated at the arch level, providing a clinically relevant unit of analysis that more accurately reflects the independent registration procedures performed during digital implant planning [14]. Second, all datasets underwent identical AI-assisted segmentation before registration, ensuring that the observed differences were attributable to the registration methods rather than variability in the segmentation process [12, 13]. Third, generalized estimating equations were used as a sensitivity analysis to account for the statistical dependence between maxillary and mandibular arches originating from the same patient, thereby confirming the robustness of the primary findings. Finally, intra-observer reliability demonstrated excellent agreement (ICC = 0.985), indicating minimal measurement error and supporting the reproducibility of the registration accuracy measurements.

Several limitations should be considered when interpreting the present findings. First, this was a retrospective single-center study, which may limit the generalizability of the results to other clinical settings and patient populations. Second, the study evaluated a single commercially available implant-planning software platform (coDiagnostiX^®^); therefore, the findings may not be directly applicable to other software systems employing different registration algorithms or AI implementations. Third, all registrations and quantitative measurements were performed by a single calibrated examiner. Although excellent intra-observer reliability was demonstrated, future studies should evaluate inter-observer reproducibility to further validate the consistency of the registration workflow. Fourthly, because previously acquired clinical CBCT datasets were retrospectively included, acquisition protocols and imaging parameters were not fully standardized across patients. Variations in field of view, voxel size, exposure settings, and reconstructed image quality may have influenced segmentation and registration performance. However, both registration methods were applied to the same CBCT dataset for each paired observation, ensuring identical imaging conditions within each comparison and thereby reducing the potential effect of acquisition heterogeneity on the between-method analysis. Nevertheless, residual confounding related to differences in CBCT acquisition cannot be excluded. Also, although the subgroup analyses provided additional insight into the influence of metallic artifact burden, these analyses were exploratory and should be interpreted cautiously until confirmed in adequately powered prospective studies. Finally, the exclusion of arches that did not meet the minimum dentition requirement for the predefined registration protocol may have introduced selection bias by limiting the representation of more severely compromised arches. Consequently, the present findings may not be directly generalizable to patients with markedly reduced residual dentition.

Future research should validate these findings through prospective multicenter clinical studies involving larger and more diverse patient populations. Comparative investigations across multiple implant-planning software platforms and AI-assisted registration algorithms are warranted to determine whether the present findings can be generalized beyond the software environment evaluated in this study. In addition, future studies should investigate the performance of AI-assisted registration in fully edentulous patients and assess its compatibility with different intraoral scanning systems. Finally, longitudinal studies evaluating the relationship between registration accuracy, surgical guide manufacturing, and implant placement accuracy would help clarify the contribution of image registration to the cumulative accuracy of computer-guided implant surgery.

## Conclusion

AI-assisted registration demonstrated significantly lower registration discrepancies than conventional point-based registration in patients presenting with metallic artifacts on CBCT images, while maintaining comparable registration duration. These findings suggest that AI-assisted registration may improve the accuracy and consistency of multimodal image registration, representing an important step within the digital implant planning workflow, particularly in clinically challenging cases affected by metallic artifacts. Although registration constitutes only one component of the cumulative digital workflow, improving registration accuracy may contribute to enhancing the overall precision of computer-guided implant surgery. Nevertheless, these findings should be interpreted within the limitations of this retrospective single-center study. Future prospective multicenter investigations involving multiple implant-planning software platforms, different AI-assisted registration algorithms, and fully edentulous clinical scenarios are warranted to confirm the generalizability and clinical applicability of the present findings.

## Data Availability

Data available on request from the authors.
